# No evidence that migratory geese disperse avian influenza viruses from breeding to wintering ground

**DOI:** 10.1371/journal.pone.0177790

**Published:** 2017-05-18

**Authors:** Shenglai Yin, David Kleijn, Gerard J. D. M. Müskens, Ron A. M. Fouchier, Josanne H. Verhagen, Petr M. Glazov, Yali Si, Herbert H. T. Prins, Willem Frederik de Boer

**Affiliations:** 1 Resource Ecology Group, Wageningen University and Research, Wageningen, The Netherlands; 2 Plant Ecology and Nature Conservation Group, Wageningen University and Research, Wageningen, The Netherlands; 3 Alterra, Centre for Ecosystem Studies, Wageningen University and Research, Wageningen, The Netherlands; 4 Department of Viroscience, Erasmus MC, Rotterdam, the Netherlands; 5 Department Biology and Environmental Sciences, Linnaeus University, Kalmar, Sweden; 6 Laboratory of Biogeography, Institute of Geography Russian Academy of Sciences, Moscow, Russia; 7 Ministry of Education Key Laboratory for Earth System Modelling, and Department of Earth System Science, Tsinghua University, Beijing, China; Lund University, SWEDEN

## Abstract

Low pathogenic avian influenza virus can mutate to a highly pathogenic strain that causes severe clinical signs in birds and humans. Migratory waterfowl, especially ducks, are considered the main hosts of low pathogenic avian influenza virus, but the role of geese in dispersing the virus over long-distances is still unclear. We collected throat and cloaca samples from three goose species, Bean goose (*Anser fabalis*), Barnacle goose (*Branta leucopsis*) and Greater white-fronted goose (*Anser albifrons*), from their breeding grounds, spring stopover sites, and wintering grounds. We tested if the geese were infected with low pathogenic avian influenza virus outside of their wintering grounds, and analysed the spatial and temporal patterns of infection prevalence on their wintering grounds. Our results show that geese were not infected before their arrival on wintering grounds. Barnacle geese and Greater white-fronted geese had low prevalence of infection just after their arrival on wintering grounds in the Netherlands, but the prevalence increased in successive months, and peaked after December. This suggests that migratory geese are exposed to the virus after their arrival on wintering grounds, indicating that migratory geese might not disperse low pathogenic avian influenza virus during autumn migration.

## Introduction

Pathogens can strongly influence host populations by reducing activity, reproduction or survival [1–3]. Many pathogens are capable of infecting more than one host species. The avian influenza viruses (AIVs), for example, are highly infectious to a wide range of wildlife, domestic animals, and humans [4–8]. In 2016, a highly pathogenic AIV (HPAIV) H5N8 was isolated from water birds in Russia, rapidly followed by isolations in India and Europe [9]. The highly infectious and fast spread of AIV has boosted research into the presence and dynamics of this pathogen in wild birds.

Based on their ability to cause disease in chickens, AIVs are characterized by two types: HPAIV, such as the one isolated in 2016, and LPAIV (low pathogenic AIV). The latter occurs more frequently in wild birds. When they are infected with LPAIV, wild birds do not show any clinical signs. Therefore, the migration of wild birds might contribute to the dispersal of LPAIV. Insights into the dynamics of LPAIV infection in migratory wild birds can help us to better understand and predict the spatial and temporal distribution of LPAIV outbreaks.

Most of what is known about the ecology of LPAIV prevalence is based on information from duck species such as mallard (*Anas platyrhynchos*). Ducks are considered the main hosts of LPAIV, because their aquatic habits facilitate transmission, spread, and persistence of LPAIV [10,11]. Previous studies from Northern Hemisphere have shown that LPAIV circulates year-round in ducks, and the infection peaks just after the breeding season when the population comprises many immunologically naive juveniles [12,13]. LPAIV prevalence typically declines after the breeding season, from as high as 60% during the post-breeding migration to as low as 0.25% during spring migration [14]. Many ducks are long-distance migrants. They encounter migratory birds from other flyways and aggregate in large numbers at stopover sites during migration. Aggregation may facilitate outbreaks of LPAIV infection because the virus can be more rapidly transmitted between individuals that occur in high density [12,15]. Long-distance migrations, encounters with other birds, and aggregation of many duck species such as mallard could provide an explanation for why AIV disperse over long distances so fast [16,17].

However, it is unlikely that all migratory waterfowl have a similar role in the dispersal of LPAIV. Other waterfowl, such as geese, might only be secondary hosts [18]. Geese may become infected after exposure to LPAIV from a primary host, but lose the LPAIV rapidly [18]. Furthermore, some species, such as Greater white-fronted goose, breed at higher latitudes than mallard. These more northerly distributed geese may be less unlikely to be exposed to LPAIV. Therefore, it has been proposed that geese are not infected with LPAIV during their breeding phase [18], which means that they might have no LPAIV infection when they start their autumn migration, and thereby, play a limited role in dispersing LPAIV from their breeding grounds along the migratory flyways. However, year-round studies of LPAIV prevalence in goose species are rare and we still lack robust evidence that LPAIV is absent in geese during part of their annual cycle.

Here, we examined prevalence of LPAIV infection in three arctic-breeding goose species, Bean goose (*Anser fabalis*), Barnacle goose (*Branta leucopsis*) and Greater white-fronted goose (*Anser albifrons*). These species have been identified as hosts of AIV [19], and they are abundant wintering goose species in central-western Europe where they aggregate in large numbers and share stopover sites and wintering grounds with high densities of a wide variety of ducks. We studied these three goose species that breed in tundra and high-latitude boreal forest wetlands from approximately June to September. After the breeding season, a large part of the population migrates to the Netherlands for overwintering. Bean geese and Greater white-fronted geese also winter in large numbers in Hungary [20]. Bean geese and Greater white-fronted geese arrive in large numbers at the Netherlands in November-December, and leave in February-March, while Barnacle geese arrive in October and leave in March [20]. During the winter of 2012–2013, there were more than 190,000 Bean geese, 750,000 Barnacle geese and 760,000 Greater white-fronted geese overwintering in the Netherlands [21].

We compared the prevalence of LPAIV infection in these three species on their breeding grounds, wintering grounds, and spring stopover sites. As these goose species might be merely secondary hosts of LPAIV, we expect them to be largely free of LPAIV on the breeding grounds, and we expect high prevalence of LPAIV on the wintering grounds, and then especially to the middle or later part of their wintering period, as the LPAIV is assumed to be transmitted only after arrival at these wintering grounds. In a previous study carried out in Snow geese (*Chen caerulescens*) from North America, prevalence of AIV infection declined in 4 out of 5 spring migrations. Therefore, we also expect an intermediate level of infection in spring migration [22]. We specifically tested 1) if LPAIV infection in all three goose species is absent on their breeding grounds; 2) if the prevalence of infection increases over time on wintering grounds; 3) and if the prevalence of infection reduces to intermediate level on spring stopover sites. As we know that factors such as age, body condition, and sex may influence prevalence of LPAIV infection in geese [18,4], we also included these factors into our analyses to compare their effects with that of temporal patterns.

## Methods

### Ethics statement

The Animal Ethics Committee of the Erasmus Medical Center (Stichting DEC Consult) approved these studies permit number 122-07-09, 122-08-12, 122-09-20, 122-10-20 and 122–11–31.

### Samples

We used data of LPAIV infection in Bean geese, Barnacle geese and Greater white-fronted geese that were collected between 2002 and 2013 within the framework of AIV surveillance programs [23]. Geese were caught and tested for AIV infection on their breeding grounds, spring stopover sites, and wintering grounds. All sampled geese were also weighed, aged and sexed, and wing length and head length were measured. All individuals were ringed before release. Samples from wintering grounds were mainly taken in the Netherlands, one of the most important wintering grounds for migratory waterfowl in central-western Europe [24]. Dutch samples (n = 8,196) were from geese caught in November-February between late 2006 and early 2013. Other samples (n = 764) were obtained from their breeding grounds, wintering grounds outside the Netherlands and spring stopover sites between 2002 and 2013. Samples from the wintering grounds outside the Netherlands were mainly from Hungary, another important wintering ground for migratory birds [24]. Samples from breeding grounds were mainly from the Kolguev Island, Russia, where the largest Russian breeding population of Greater white-fronted geese and a significant proportion of the Barnacle geese can be found. Samples from spring stopover sites were mainly from Kologriv, Russia, an important spring stopover site of migratory geese. The breeding grounds and the spring stopover sites are located on the flyway of the Greater white-fronted geese population that winters in the Netherlands [25]. The sampled geese were caught by means of live decoys, mechanical clap-nets, cannon nets, or standing nets. A small part of our data had been analysed in previous studies [18,26].

### Virus detection

Sterile cotton swabs were used to collect cloaca, throat or combined cloaca-throat samples from each individual goose. Samples were stored in transport medium at 4°C for maximally two weeks until transported to the laboratory, where samples were stored at -80°C until testing [18]. RNA was isolated by the MagnaPure LC system with the Magna-Pure LC total nucleic acid isolation kit (Roche Diagnostics, Almere, the Netherlands), and the AIVs were detected by a generic real-time reverse transcriptase PCR (RRT-PCR) assay targeting the matrix (M) gene (M RRT-PCR). Amplification and detection were performed on an ABI 7700 machine with a TaqMan EZ RT-PCR core reagents kit (Applied Biosystems, Nieuwerkerk aan den IJssel, the Netherlands) and 20 μl of RNA eluate in an end volume of 50 μl. A more detailed method description can be found in previous studies [18,27].

### Data analysis

The prevalence of infection was expressed as the percentage of positive samples in each group of interest (e.g., species or season). As the sample sizes from outside the Netherlands were low, we used straight-forward proportion tests on the total number of samples from each location to examine if prevalence of infection differed among breeding grounds, spring stopover sites, wintering grounds in Hungary, and wintering grounds in the Netherlands.

To determine if the prevalence of infection on the wintering grounds was significantly lower directly after the arrival of migratory geese compared to later months, we only used the dataset from the Netherlands. Because sample size varied dramatically among years, we furthermore restricted our analysis to the years with more than 200 samples. Sample size in each year is shown in S1 Table.

We constructed Generalized Linear Models (GLMs) for each species, assuming a binomial error distribution, and using a logit link function. The response variable was infection status (binomial), and the predictor variables included year (categorical), month (categorical), sex, age, body condition, interaction between year and age, and interaction between year and body condition. Body condition index was calculated as described in a previous study [28]. We used the first principle component (PC1) of a PCA analysis of wing length and head length as an index of body size. Next, the index of body size and log-transformed body weight were included in an ordinary least squares (OLS) regression to calculate the residuals. The residuals were used as an index of body condition, and the individual with a greater and positive residual was considered to have a better body condition than those with a lower residual [29].

We used a multi-model inference approach to determine the best set of models to describe the variation in prevalence of infection. In total, 52 models were ranked with ascending AICc (Akaike information criterion corrected for small sample size) scores for each species. The top model sets were selected, using the criterion AICc<2 [30]. To account for model selection uncertainty, model averaging was carried out using the full-model method [31]. For Barnacle geese and Greater white-fronted geese, we chose the top model to predict the prevalence of infection and examine the effect of month. For Bean geese, we chose the second model form the rank because it includes the variable month. Tukey’s post hoc test was used to test for differences in prevalence among years and months. Effect of age on prevalence of LPAIV infection in Greater white-fronted goose was examined in each year separately. All statistical and modelling analyses were carried out in R 2.11.0 [32].

## Results

### LPAIV infection in different parts of the flyway

Out of the 268 samples collected on breeding grounds and 297 on spring stopover sites, none tested positive for LPAIV. LPAIV infection, however, was detected in samples from wintering grounds in both the Netherlands and Hungary (Table 1). Proportion test confirmed that prevalence of infection on the wintering grounds (both in Hungary and the Netherlands) was significantly higher than that on the breeding grounds or spring stopover sites (df = 3, Z score = 8.69, P<0.001).

**Table 1:** Prevalence of LPAIV infection and sample sizes (N positive/N total) of Bean goose (*Anser fabalis*), Barnacle goose (*Branta leucopsis*) and Greater white-fronted goose (*Anser albifrons*) at breeding grounds, spring stopover sites and wintering grounds in Hungary and the Netherlands.

Locations	Bean goose	Barnacle goose	Greater white-fronted goose	Total
breeding grounds	0 (0/22)	0 (0/9)	0 (0/237)	0 (0/268)^a^
spring stopovers	0 (0/10)	0 (0/2)	0 (0/285)	0 (0/297)^a^
wintering grounds (Hungary)	27% (6/22)	0 (0/7)	11% (18/170)	12% (24/199)^b^
wintering grounds (Netherlands)	2% (12/508)	12% (173/1404)	13% (798/6374)	12% (983/8286)^b^

a and b refer to the statistical differences at *α* = 0.05 (proportion test, df = 3, Z score = 8.69, P<0.001).

### Infection variation on the wintering grounds

**Fig 1:**
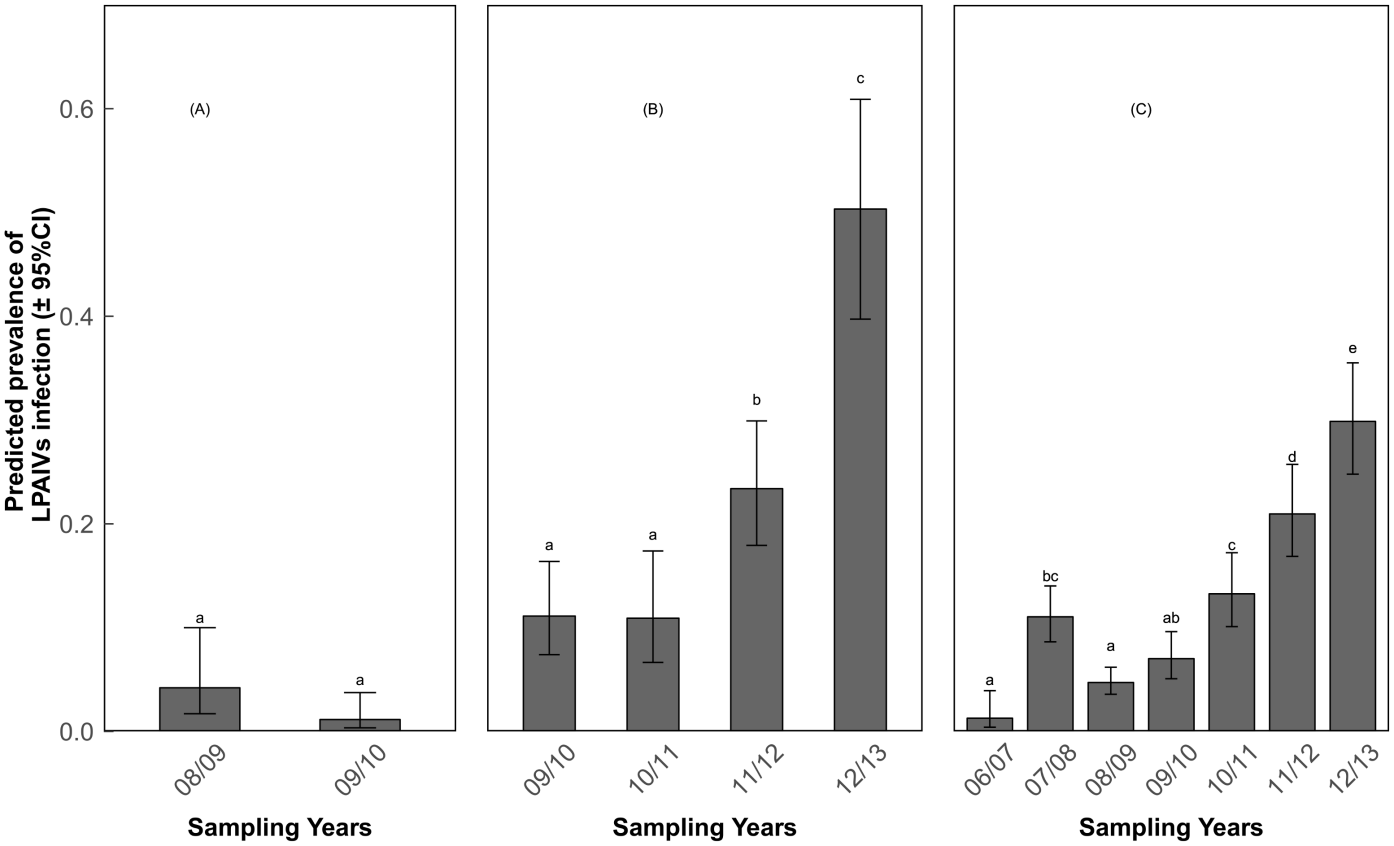
Predicted prevalence of LPAIV infection (±95% confidence interval) in each year for three species, separately. (A) Bean geese; (B) Barnacle geese; (C) Greater white-fronted geese. a, b and c refer to the statistical difference at α = 0.05.

**Fig 2:**
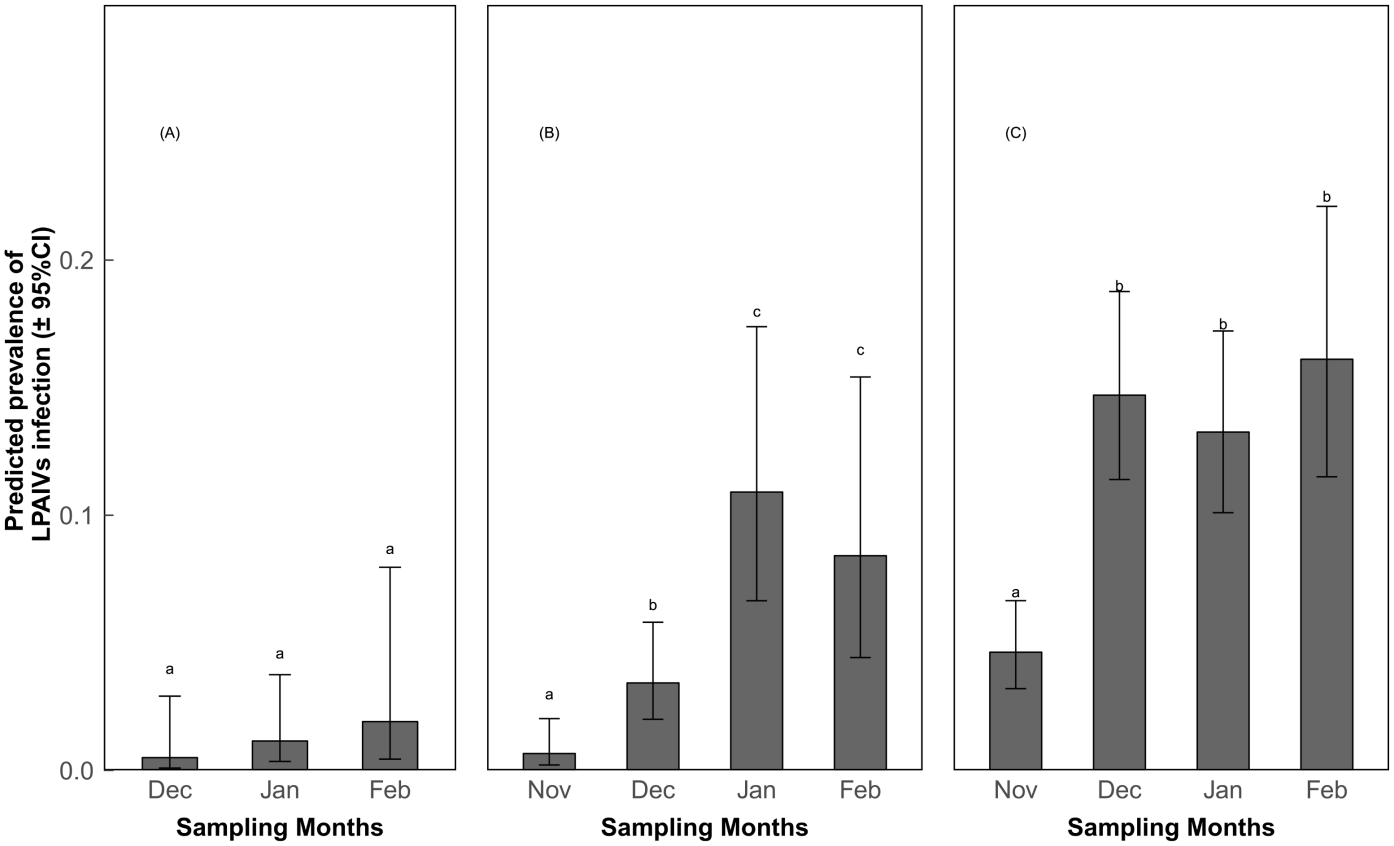
Predicted prevalence of LPAIV infection (±95% confidence interval) in each month for three species, separately. (A) Bean geese; (B) Barnacle geese; (C) Greater white-fronted geese. a, b and c refer to the statistical difference at α = 0.05.

**Fig 3:**
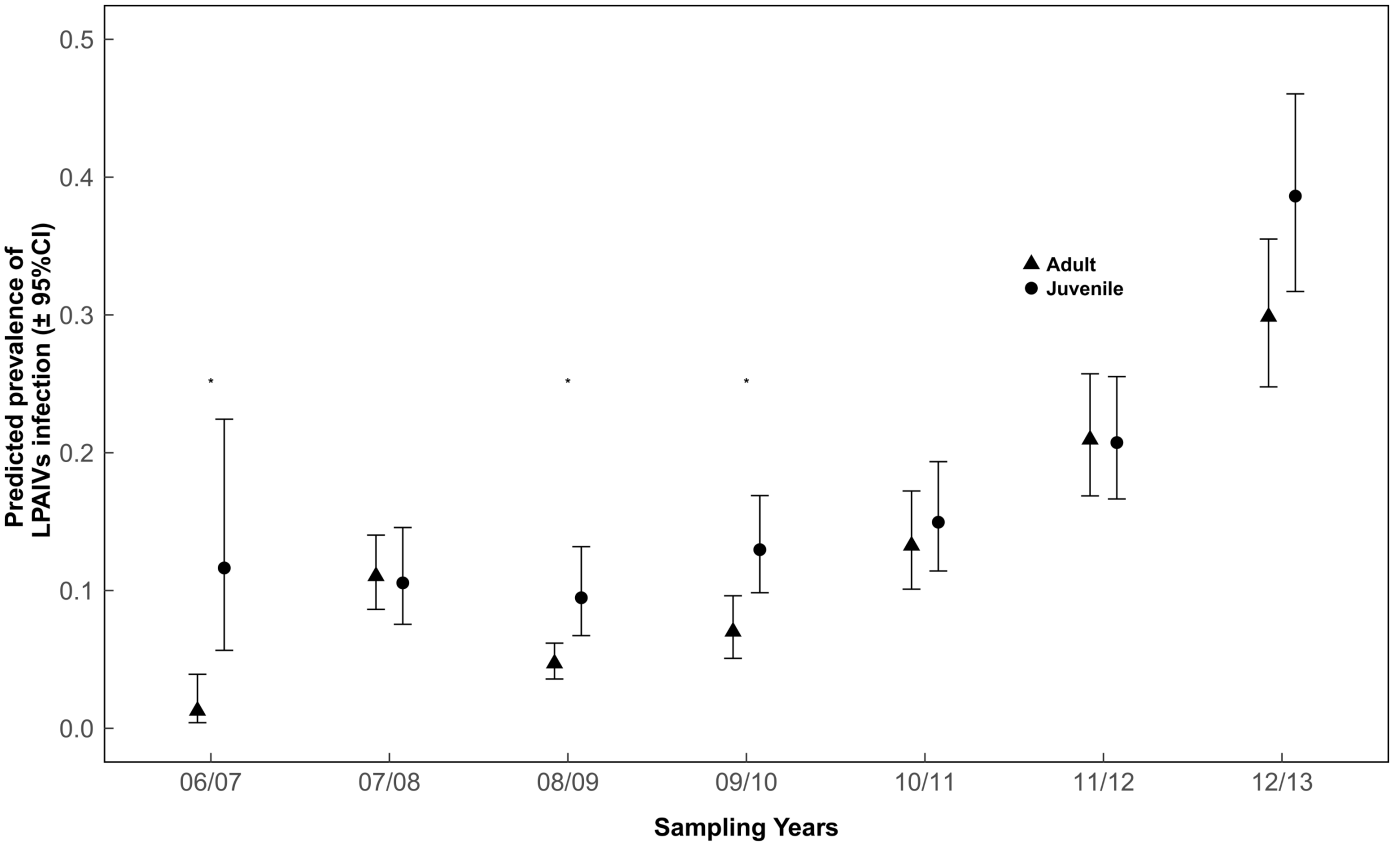
Predicted prevalence of LPAIV infection (±95% confidence interval) over years in adult and juvenile Greater white-fronted geese.

The multi-model inference approach revealed that prevalence of LPAIV infection was associated with different variables for different species. For Bean geese, only year was included in the top model set (Table 2). We included the variable month in the model to examine the variation of prevalence over months. The subsequent tests showed that the prevalence of LPAIV infection was slightly higher in 2008–2009 compared to 2009–2010 (Fig 1A; Z value = -1.94, P = 0.052), and the prevalence slightly increased over the months (Fig 2A; Z value>0.77, P>0.267), but the differences were not statistically significant at the α = 0.05 level. For Barnacle geese, year, month, age, body condition and the interaction between year and body condition were included in the top model (Table 2). The prevalence in the last two years was higher than in the previous years, and peaked in 2012–2013 (Fig 1B; Z value>3.12, P<0.01). Prevalence of LPAIV infection increased dramatically over the months with the highest values in January and February (Fig 2B; Z value>2.92, P<0.016). For Greater white-fronted geese, year, month, age, body condition, the interaction between year and body condition, and the interaction between year and age were included in the top model. Similar to Barnacle goose, Greater white-fronted geese had a higher prevalence in the last two years (Fig 1C; Z value>3.44, P<0.01). Prevalence of LPAIV infection in December-February was higher than in November (Fig 2C; Z value>7.37, P<0.001). The differences in prevalence of LPAIV infection between juvenile and adult Greater white-fronted geese differed among sampling years, as juvenile birds had higher prevalence than adults in 2006–2007 (Fig 3; Z = -3.47, P = 0.005), 2008–2009 (Z = -2.87, P = 0.004) and 2009–2010 (Z = -3.13, P = 0.002), but not in others (Z value>-1.95, P>0.05).

**Table 2:** Summarized results of the multi-model inference approach.

Species	model	year	month	sex	age	bc	Year×bc	year×age	ω_m_	ΔAICc
Bean goose	1	-1.29							0.44	0.00
	2	-1.33	+						0.22	1.41
	3	-1.28		0.27					0.18	1.81
	4	-1.25			0.29				0.17	1.83
	β	-1.29	+	-1.29	0.05					
	ω_p_	1.00	0.21	0.18	0.17					
Barnacle goose	1	+	+		0.29	0.64	+		0.44	0.00
	2	+	+			0.62	+		0.37	0.32
	3	+	+		-0.33	0.61	+	+	0.19	1.71
	β	+**	+**		0.07	0.63	+	+		
	ω_p_	1.00	1.00		0.63	1.00	1.00	0.19		
Greater white-fronted goose	1	+	+		-2.32	0.71	+	+	0.73	0.00
	2	+	+	-0.01	-2.32	0.71	+	+	0.27	1.98
	β	+***	+***	-0.01	-2.32***	0.71	+	+*		
	ω_p_	1.00	1.00	0.27	1.00	1.00	1.00	1.00		

The candidate models were ranked in order of increasing difference of AICc (ΔAICc<2); The parameter estimates for males as compared to zero for females. The parameter estimates for adults as compared to zero for juveniles. The parameter estimates in each candidate model are given in columns. β indicates the averaged estimates. ω_p_ indicates the relative importance. ω_m_ indicate the probability that the model is the best approximating model in the set. bc refers to body condition. + indicates this factor variable was included in the model. asterisks refer to the statistical difference (*, P<0.05; **, P<0.01; ***, P<0.001).

## Discussion

The large distances covered by migratory geese and the mixing of different flyway populations during parts of their annual cycle cause concerns about the dispersion of AIV across continents. We found no LPAIV infection in three goose species on their breeding grounds or spring stopover sites. The prevalence of LPAIV infection was low just after their arrival on wintering grounds in the Netherlands but increased during successive months to peak after December. These results suggest that the studied migratory goose species are free of LPAIV infection on their high-latitude breeding grounds, and only become infected after exposure to LPAIV on their wintering grounds.

The lack of any LPAIV infection in the three goose species on their breeding grounds (Table 1) is in line with a previous study that found geese might have no LPAIV infection during autumn migration [22,33]. Furthermore, a study on LPAIV infection of pink-footed geese (*Anser bachyrhynchus*) over their flyway gave a similar result, namely infection was only found on their wintering grounds [34]. These findings indicate that migratory geese can lose LPAIV in some phases of their annual cycle, and strongly suggest that migratory geese do not carry LPAIV from their breeding grounds to their wintering grounds. This contrasts with ducks such as mallards, which can preserve LPAIV in the population over their entire annual cycle [35]. Compared to ducks, geese are less restricted to wetland habitat, and they mainly forage on land and defecate in compact droppings. These different traits reduce the chance for faecal-oral transmission [18], and reduce environmental transmission, which is important for LPAIV to persist in a waterfowl population [36–38]. This could be one factor explaining why geese, but not ducks, are free of LPAIV during part of their annual cycle.

Migratory waterfowl are generally considered asymptomatic carriers of LPAIV because they do not show serious disease signs when infected. Consequently, they are potentially important in dispersing LPAIV over long distances [39]. If migratory waterfowl carry LPAIV during autumn migration, prevalence of LPAIV infection should have been stable or decreased after their arrival on the wintering grounds. However, LPAIV prevalence in the examined geese was low upon their arrival in October-November (Fig 2). Only after November, did prevalence of infection increase in wintering geese. This suggests that migratory geese were exposed to LPAIV that circulated on wintering grounds. This is consistent with the idea that migratory birds more likely amplify local LPAIV infection, because they may not be immune to a locally circulating virus and may have reduced immunocompetence because of physiological costs of migration [40,41].

Although Barnacle and Greater white-fronted geese had a relatively high prevalence of infection in February (Fig 2B and 2C), they did not show any infection on spring stopover sites in Russia (Table 1). This refutes our supposition that the prevalence of LPAIV infection is intermediate on spring stopover sites. In Canada geese (*Branta canadensis*), virus shedding after infection with HPAIV takes up to 6 days [42]. Assuming Barnacle and Greater white-fronted geese have a similar shedding period to that of Canada geese, they had plenty of time to recover from the infection during the spring migration. The absence of LPAIV infection, especially in Greater white-fronted geese, on spring stopover sites suggests that these geese do not maintain the virus within their own population and that they are not being exposed to new LPAIV during spring migration. However, sample sizes from spring stopover sites were low, and analyses based on larger sample sizes are required to confirm this tentative conclusion.

Variations of LPAIV infection among years are commonly reported in wild birds [13,18,41]. In our study, prevalence of LPAIV infection significantly increased in the last two years in Barnacle geese and Greater white-fronted geese (Fig 1B and 1C). High prevalence of LPAIV infection is frequently associated with a large proportion and number of juveniles in the population, because they are immunological naive [12]. However, the proportions of juvenile Barnacle geese and juvenile Greater white-fronted geese in the last two years were not consistently higher than those in previous years [21,43]. Moreover, prevalence of infection in Greater white-fronted geese did not differ between juveniles and adults in these last two years (Fig 3; P>0.05). Therefore, the proportion of immunological naive juveniles in the population alone cannot explain the variations in the prevalence of LPAIV infection in this study, which also begs for further study.

Our findings, which are largely based on data from Greater white-fronted geese, suggest that migratory geese are free of LPAIV infection before their autumn migration, and exposed to LPAIV after their arrival on the wintering grounds. It indicates that migratory geese are secondary hosts of LPAIV, that they are free of LPAIV infection during certain parts of their annual cycle. Therefore, there is no evidence that migratory geese disperse AIV over their migration flyways. More likely, migratory geese arriving and aggregating on their wintering grounds amplify the infection of local LPAIV, instead of introducing novel strains from afar.

## Supporting information

S1 Table Sample size of each species in successive years in the Netherlands. (DOCX)

S2 Table Mean estimated geese population size in the Netherlands from July 2006-May 2013. The number of geese are averaged with the actual counting of each month from 2006/2007 to 2012/2013; All the data are from Sovon reports Watervogels in Netherland in 2006/2007, 2007/2008, 2008/2009, 2009/2010, 2010/2011, 2011/2012 and 2012/2013 (https://www.sovon.nl/sovonrapporten).
(DOCX)

S3 Table Sample collection and virus detection for avian influenza virus from three samples goose species. (XLSX)
