# Supplementary material for: No evidence that migratory geese disperse avian influenza viruses from breeding to wintering ground
Source: PLoS One. 2017 May 18;12(5):e0177790. doi: 10.1371/journal.pone.0177790 (PMC5436700; doi:10.1371/journal.pone.0177790)
Supplement: S1 Table — (DOCX) [file pone.0177790.s001.docx]

S1 Table Sample size of each species in successive winters in the Netherlands.

| Winter | Bean goose | Barnacle goose | Greater white-fronted goose |
| --- | --- | --- | --- |
| 2006-2007 | 71 | 43 | 282 |
| 2007-2008 | 91 | 171 | 1,050 |
| 2008-2009 | 235 | 61 | 1,522 |
| 2009-2010 | 274 | 380 | 987 |
| 2010-2011 | 62 | 372 | 811 |
| 2011-2012 | 45 | 385 | 1,058 |
| 2012-2013 | 13 | 267 | 664 |
| Total | 508 | 1,404 | 6,284 |
