# Supplementary material for: No evidence that migratory geese disperse avian influenza viruses from breeding to wintering ground
Source: PLoS One. 2017 May 18;12(5):e0177790. doi: 10.1371/journal.pone.0177790 (PMC5436700; doi:10.1371/journal.pone.0177790)
Supplement: S2 Table — The number of geese are averaged with the actual counting of each month from 2006/2007 to 2012/2013; All the data are from Sovon reports Watervogels in Netherland in 2006/2007, 2007/2008, 2008/2009, 2009/2010, 2010/2011, 2011/2012 and 2012/2013 (https://www.sovon.nl/sovonrapporten). (DOCX) [file pone.0177790.s002.docx]

S2 Table Mean estimated goose population size in the Netherlands from July 2006-May 2013.

| Species | July | Aug. | Sept. | Oct. | Nov. | Dec. | Jan. | Feb | Mar. | Apr. | May | June |
| --- | --- | --- | --- | --- | --- | --- | --- | --- | --- | --- | --- | --- |
| Bean goose | 33 | 1 | 15 | 13,660 | 87,663 | 335,280 | 176,431 | 127,608 | 662 | 24 | 1 | 2 |
| Barnacle goose | 15,853 | 14,893 | 30,172 | 118,412 | 314,670 | 420,221 | 503,470 | 549,122 | 458,145 | 262,323 | 102,150 | 8,665 |
| Greater white-fronted goose | 102 | 129 | 1,645 | 223,681 | 616,648 | 655,524 | 764,731 | 667,125 | 366,123 | 3,658 | 102 | 42 |
| Total | 15,988 | 15,023 | 31,832 | 355,753 | 1,018,981 | 1,411,025 | 1,444,632 | 1,343,856 | 824,930 | 266,005 | 102,253 | 8,708 |
